# Genome size in arthropods; different roles of phylogeny, habitat and life history in insects and crustaceans

**DOI:** 10.1002/ece3.3163

**Published:** 2017-06-22

**Authors:** Kristian Alfsnes, Hans Petter Leinaas, Dag Olav Hessen

**Affiliations:** ^1^ Department of Biosciences University of Oslo Oslo Norway; ^2^ Department of Molecular Biology Norwegian Institute of Public Health Oslo Norway

**Keywords:** crustaceans, *C*‐value, ecology, evolution, insects, life history, temperature‐size‐rules

## Abstract

Despite the major role of genome size for physiology, ecology, and evolution, there is still mixed evidence with regard to proximate and ultimate drivers. The main causes of large genome size are proliferation of noncoding elements and/or duplication events. The relative role and interplay between these proximate causes and the evolutionary patterns shaped by phylogeny, life history traits or environment are largely unknown for the arthropods. Genome size shows a tremendous variability in this group, and it has a major impact on a range of fitness‐related parameters such as growth, metabolism, life history traits, and for many species also body size. In this study, we compared genome size in two major arthropod groups, insects and crustaceans, and related this to phylogenetic patterns and parameters affecting ambient temperature (latitude, depth, or altitude), insect developmental mode, as well as crustacean body size and habitat, for species where data were available. For the insects, the genome size is clearly phylogeny‐dependent, reflecting primarily their life history and mode of development, while for crustaceans there was a weaker association between genome size and phylogeny, suggesting life cycle strategies and habitat as more important determinants. Maximum observed latitude and depth, and their combined effect, showed positive, and possibly phylogenetic independent, correlations with genome size for crustaceans. This study illustrate the striking difference in genome sizes both between and within these two major groups of arthropods, and that while living in the cold with low developmental rates may promote large genomes in marine crustaceans, there is a multitude of proximate and ultimate drivers of genome size.

## 1 INTRODUCTION

Genome size varies greatly both within and among various taxonomic levels of plants and animals, and a number hypotheses for the selective drivers of either small or large genome size have been proposed (Cavalier‐Smith, 1978; Gregory, 2005; Lynch & Walsh, 2007). Several processes may lead to genome enlargement or genome streamlining, which subsequently may affect a number of fitness‐related traits (Petrov, 2001), such as gene activity and cell size as well as metabolic rate, growth and body size, and thereby being subject to selection (Hessen, Daufresne, & Leinaas, 2013). Over evolutionary time these processes have led to clade‐specific differences in genome size at higher taxonomic levels as well as distinct variations among related species and even conspecific populations (i.e., in snapping shrimps in Jeffery, Hultgren, Chak, Gregory, and Rubenstein (2016a). Consequently, disentangling patterns of genome size variations at different taxonomic levels is highly relevant both to ecological and evolutionary theory.

Two principally different mechanisms may have major impact on genome size: whole‐genome duplication events (polyploidization) and accumulation of noncoding elements, first and foremost transposable–and repetitive elements (Dufresne & Jeffery, 2011; Lynch & Walsh, 2007). Duplication events occur suddenly and stochastically in the genome, and may include partial or whole‐genome duplication. Compared to the duplications, accumulation of noncoding elements is a more gradual process, repeatedly adding new elements to the genome, and thus a priori yield less distinctive phylogenetic footprints (Brookfield, 2005; Feschotte, 2008; Feschotte & Pritham, 2007; Kidwell & Lisch, 2001).

Gene duplication could be beneficial by increasing the expression of fitness‐promoting gene products, as has been suggested for endopolyploidy, that is increased ploidy levels of specific tissues (Neiman, Beaton, Hessen, Jeyasingh, & Weider, 2015), but may also be nonadaptive. Potential benefits of increased accumulation of non‐protein‐coding elements are even less evident, despite the fact that genomes of most eukaryotic organisms are dominated by such elements. Whether the noncoding elements should be seen as “junk” or “selfish” DNA (Dawkins, 1976; Orgel & Crick, 1980) or may serve fitness‐promoting purposes at the organism level, is a matter of heated debate (Brunet & Doolittle, 2015; Graur et al., 2013). A direct cost of large genomes is the increased requirements for scarce and limiting elements such as nitrogen and phosphorus, which may be a drawback in nutrient scarce environments (Guignard et al., 2016; Hessen & Persson, 2009; Lewis, 1985). Bulky genomes are also costly in terms of slowing down cell‐division, growth rates, and metabolism (Gregory, 2005; Kozłowski, Konarzewski, & Gawelczyk, 2003), implying reduced growth‐ and development rates (Gregory & Johnston, 2008; White & McLaren, 2000; Wyngaard, Rasch, Manning, Gasser, & Domangue, 2005). This in turn is likely to increase adult body size and generation time (voltinism), which may affect fitness positively or negatively depending on the environment. Finally, population size could serve as a means of regulating genome size, where large populations better could counteract drift and the mutational burden imposed by transposon proliferation (Lynch, 2010; Lynch & Walsh, 2007).

In some invertebrate phyla, there is a clear positive relationship between genome size and body size (Gregory, 2001; Hessen, Ventura, & Elser, 2008). This has been documented in amphipods and copepods in colder waters (Angilletta, Steury, & Sears, 2004; Atkinson, 1994; Leinaas, Jalal, Gabrielsen, & Hessen, 2016; Timofeev, 2001), and in deepwater crustaceans (Jeffery, Yampolsky, & Gregory, 2016b; Rees, Belzile, Glemet, & Dufresne, 2008; Timofeev, 2001). These findings have been attributed low temperature and low metabolic rate. However, there can also be considerable variability in genome size among organisms of similar body size (Gregory, Hebert, & Kolasa, 2000; Leinaas et al., 2016) and even at the intraspecific level (McLaren, Sévigny, & Frost, 1989). The fact that different species or taxa display different patterns of genome–body size relation suggests the result of several processes, ranging from micro‐evolutionary adaptation to current environments, to the maintenance of phylogenetic ancient patterns (which may or may not reflect adaptive traits). Differences in genome size have also been linked with developmental complexity (Gregory, 2002), such as hemimetabolous vs. holometabolous development in insects (Gregory, 2005).

Patterns of genome size variation among organisms at different levels of taxonomic relatedness could elucidate causalities and implications, and help to distinguish between evolutionary drivers at various timescales (Gregory, 2005). To address these issues, we investigate here the genome size of the two major arthropod groups: the crustaceans (Subphylum: Crustacea) and the insects (Class: Insecta) based on publicly available data. Both focal groups include species with widely different life strategies across a wide range of distribution that allow for identification of common traits and drivers for small versus large genomes within and between groups. Insects are almost exclusively terrestrial, at least in the adult stage, while crustaceans by and large are aquatic. This has profound implications for the environmental drivers and life history strategies of the groups. In particular, patterns of seasonal and diurnal temperature variations will differ fundamentally between terrestrial and aquatic systems. This offers the possibility to evaluate genome size patterns of these groups in relation to their highly contrasting environments. After examining the phylogenetic distribution of the genome size, we subsequently screened for environmental effects using observational data as proxies for the organisms’ habitat.

## 2 METHODS

We obtained a comprehensive list of crustacean and insect genome size (pg haploid DNA per cell or 1C) from the Genome Size Database (Gregory, 2001). A few species were represented in the database with multiple entries, in this study; we present an average C‐value for each species. Species names were cross‐referenced to the NCBI taxonomy database using R v3.1.3 with the *taxize* package v0.6.6. Dendrograms were obtained with *phyloT* (http://phylot.biobyte.de/index.html) using the lineage information from NCBI taxonomy.

Observational data of the species were obtained from the gBif database using R with the *rgbif* package v0.8.0 and the *spocc* package v0.4.0. From gBif we obtained for each species; observations of the maximum absolute latitude (the most northern or southern extent) (in degrees) (MAL), maximum depth (in meters, crustaceans only) (MDE) and maximum elevation (in meters, insects only) (MEL). Maximum organism size (in millimeters) (MOS) for a selection of crustaceans was obtained from Hessen and Persson (2009). Habitat (HAB) for crustaceans was defined as *freshwater*,* marine,* or *terrestrial*, and obtained from the WoRMS database (www.marinespecies.com) and the Encyclopedia of Life database (www.eol.com). For insects, we distinguished between *hemimetabolous* and *holometabolous* development (our dataset also included two *ametabolous* species) (DEV). The obtained data were uploaded to iTOL (http://itol.embl.de/) for visualization.

Taxonomical information was obtained for a subset of the annotated species from the Genome Size Database (62% for crustaceans and 74% for insects, Table 1). Habitat (HAB) for crustaceans and insect developmental mode (DEV) was identified for all species included in this study (Table 1). Observational data: maximum absolute (most northern or southern) latitude (MAL), maximum depth (MDE) for crustaceans, and maximum elevation (MEL) for insects, were found for a subset of the species obtained with taxonomical information (MAL: 95%, MDE: 36% for crustaceans, MAL: 74%, MEL: 55% for insects, Table S2). Crustacean body sizes (MOS) were found from existing literature and a subset of matching species to the dataset included in this study was obtained (60%, Table 1).

**Table 1:** Sample overview, Blomberg's *K* and Pagel's λ

	*n*	Average	Range	*K*	λ
Crustaceans					
C‐values (pg)^a^	293	4.9	0.1−64.6	NA	NA
C‐values (pg)^b^	182	5.3	0.1−50.9	0.65**	0.99***
MOS (mm)	110	110.8	0.6−1,260.0	0.46**	0.59***
MAL (°)	171	53.3	7.3−90.0	0.44**	0.71***
MDE (m)	153	305.5	0.5−5,422.5	0.69**	0.99***
HAB	182	NA	NA	4.75***	0.99***
Insects					
C‐values (pg)^a^	793	1.2	0.1−16.9	NA	NA
C‐values (pg)^b^	586	1.1	0.1−16.9	1.47**	0.99***
MAL (°)	432	50.2	7.0−86.0	0.34*	0.74***
MEL (m)	323	1,957.6	47.5−3482.5	0.23	0.17***
DEV	586	NA	NA	17.39***	0.99

*p < 0.05, **p < 0.01, ***p < 0.001. NA = Not applicable.

^a^C‐values (pg DNA) from every species obtained from Genome Size Database.

^b^C‐values (pg) only from species with obtained taxonomic information.

Regular linear optimal least square models (OLS/lm) were calculated using R v3.1.3 with the *rms* package v5.1.0, phylogenetic generalized least squares (PGLS) was performed using the *caper* package v0.5.2. The PGLS algorithm does not allow for the unresolved polytomies (where an internal node of a cladogram has more than two immediate descendants–sister taxa) present in our dendrograms, the polytomies were removed using R with the *phytools* package 0.5.0 (using [multi2di] with random allocation–adding minute differences to the sister taxa to allow for PGLS). The *phytools* package was also used for the Blomberg's *K* (Blomberg, Garland, & Ives, 2003) and Pagel's λ (Pagel, 1999). These allow for two different measures of the phylogenetic correlation of variables; Blomberg's *K* is a variance ratio (variables are independent of the phylogeny when *K *< 1, and dependent of the phylogeny when *K *≥ 1), and Pagel's λ is a scaling parameter and given in a range from 0 (the variation of a variable is completely different from the phylogenetic pattern) to 1 (the variation of a variable is similar to the phylogenetic pattern).

## 3 RESULTS

**Figure 1:**
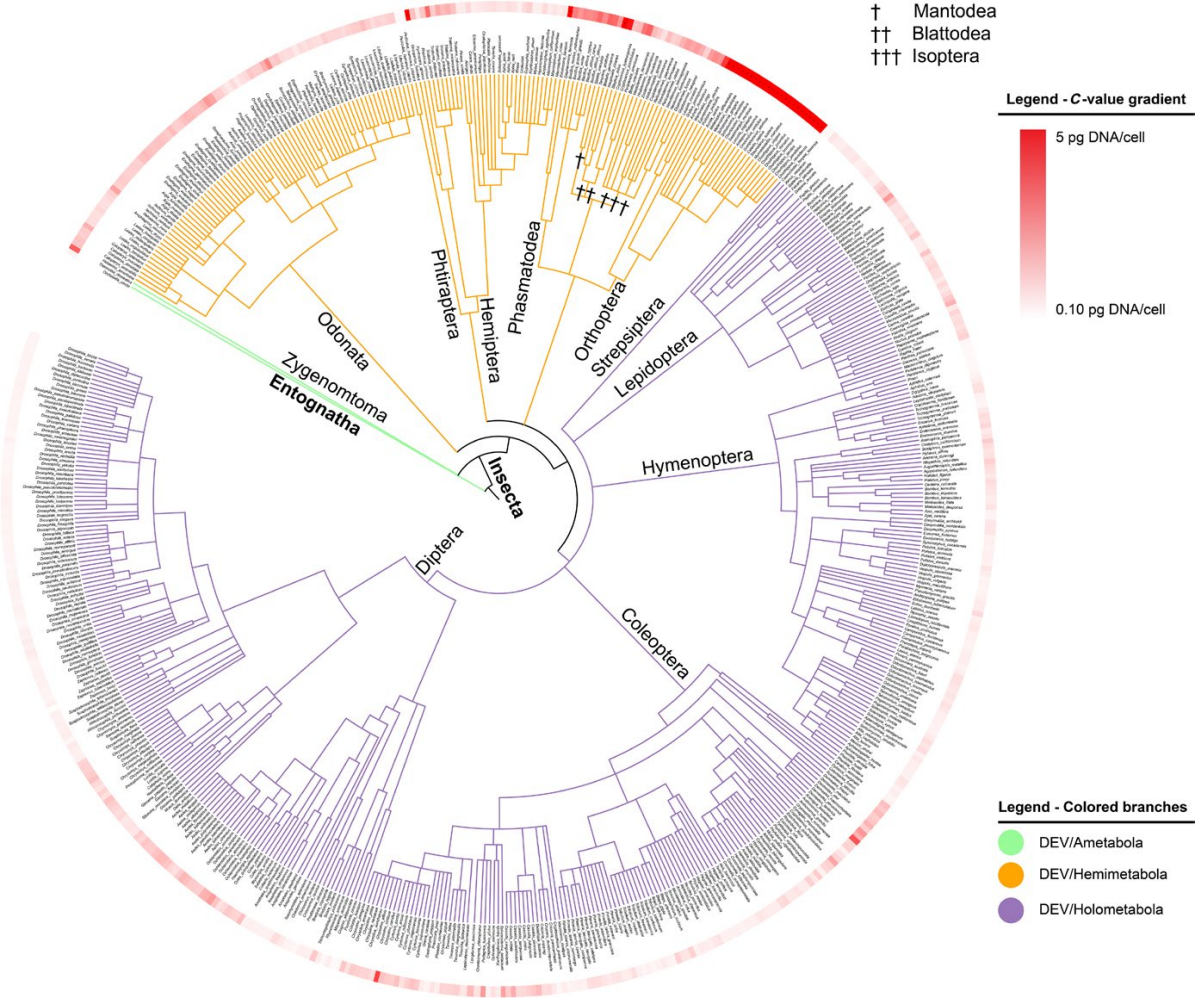
Dendrogram of insects with known C‐values (*n *= 586). C‐values (maximum shown value 5 pg DNA/cell) shown in red gradient (minimum/light red = 0.10 pg DNA/cell, maximum/dark red = 5 pg DNA/cell). C‐values above the set threshold are marked with asterisk (*); specific C‐values may be retrieved from the Table S2. Branches colored according to mechanism of DEV (green = Ametabola, orange = Hemimetabola, and purple = Holometabola). Class (in bold), order, and other notable groups (Sc = subclass) shown next to branches

**Figure 2:**
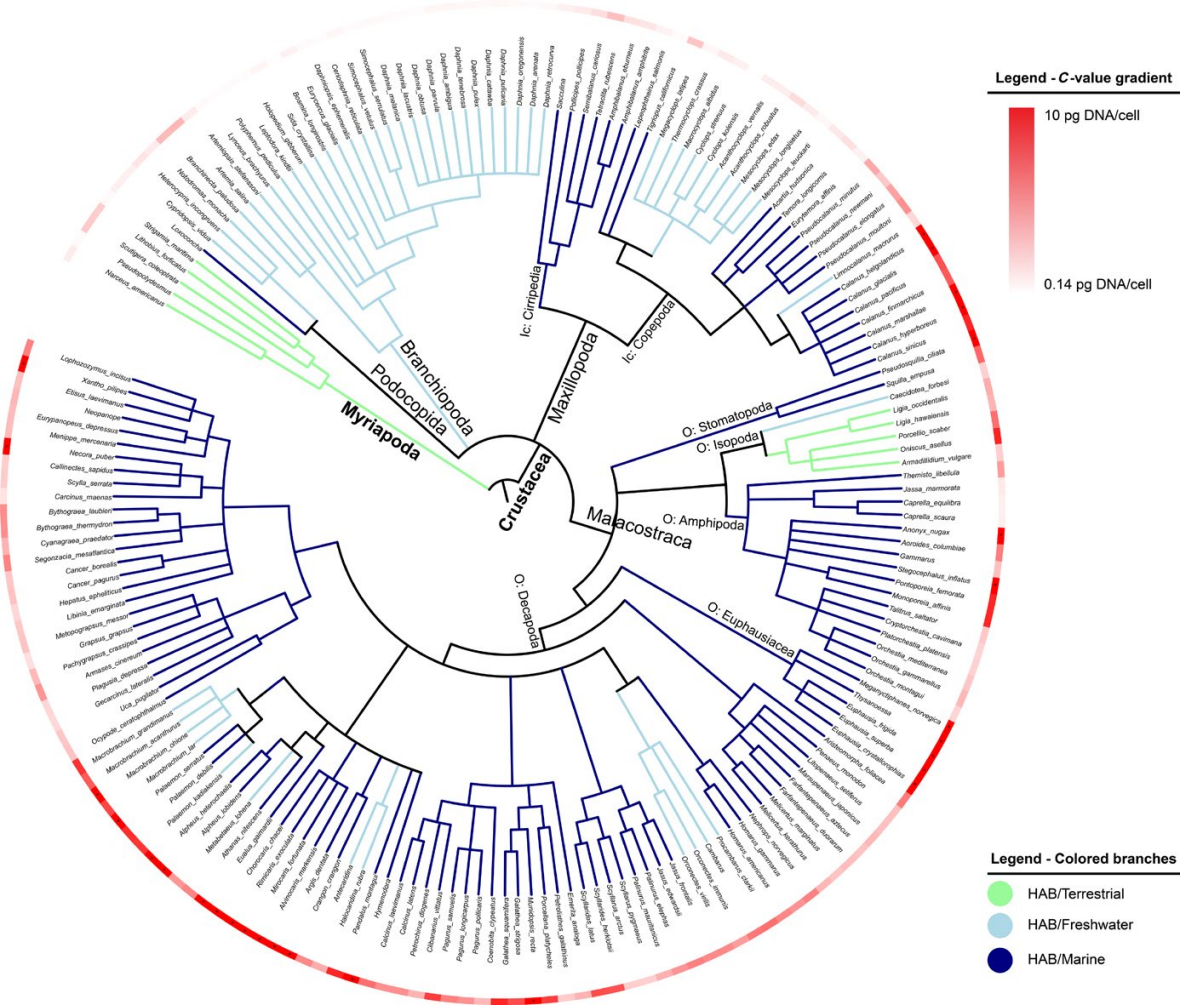
Dendrogram of crustaceans with known C‐values (*n *= 182). C‐values (maximum shown value 10 pg DNA/cell) shown in outer circle in red gradient (minimum/light red = 0.14 pg DNA/cell, maximum/dark red = 10 pg DNA/cell). C‐values above the set threshold are marked with asterisk (*); specific C‐values may be retrieved from the Table S1. Branches colored according to habitat (green = terrestrial, light blue = freshwater, and dark blue = marine). Subphylum (in bold), class, and other notable groups (Ic = infraclass, O = order) shown next to branches

Taxonomy‐based dendrograms were constructed for all crustaceans and insects for which genome size could be obtained from the database (Figures 1 and 2). For all species, the genome sizes are visualized by a red circle, where darker colors correspond with larger genome sizes. In insects, the great difference in genome size between Hemimetabola and Holometabola is clearly seen in Figure 1. As a result, Blomberg's K showed a clear phylogenetic dependence (*K* > 1) of genome size in this group (Table 1). By comparison, the crustaceans showed a very different pattern (Figure 2). Genome size varied much more at lower phylogenetic levels, which is reflected by much lower Blomberg's *K* (Table 1). Figure 2 illustrates distinct phylogenetic patterns even in this group, where some taxa, such as calanoid copepods, krill (Euphausiacea), and shrimps (Caridea) show systematically larger genomes than others, while Branchiopoda and cyclopoid copepods had systematically very small genomes.

**Figure 3:**
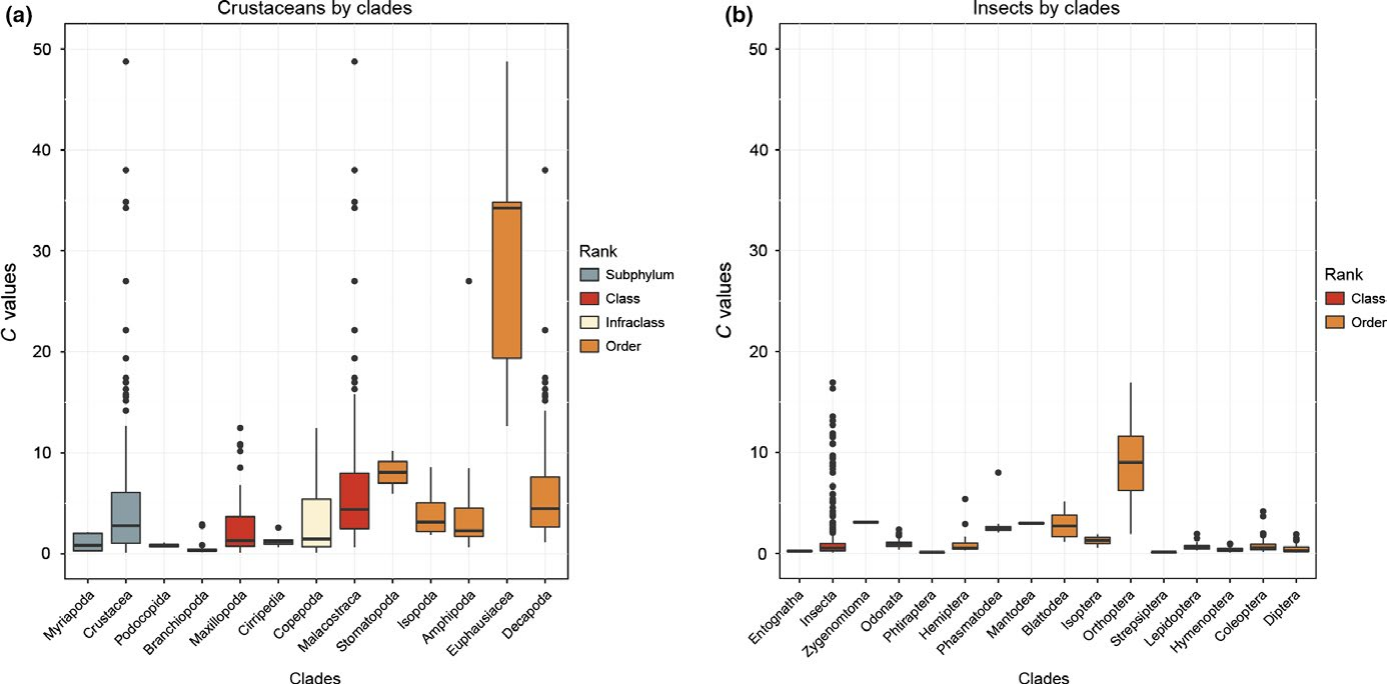
C‐values by clades in crustaceans (a) (with representatives from the outgroup Subphylum Myriapoda as represented in Figure 2). The class Maxillopoda show the combined C‐values of the infraclasses Cirripedia and Copepoda, and the class Malacostraca show the combined C‐values of the orders Stomatopoda, Isopoda, Amphipoda, Euphausiacea, and Decapoda, and insects (b) (with representatives from the outgroup Class Entognatha as represented in Figure 1)

In both the insects and crustaceans genome variations at lower phylogenetic levels are likely, at least partly, to reflect specific adaptations. Groups like isopods, amphipods, and several decapod taxa show striking variability that appears disconnected from phylogeny. For the insects, the diminutive genomes in the parasitic *Pediculus humanus* stand out against the generally large genomes of the other species with hemimetabolous development (Figure 1). The clade‐specific genome size variations are shown in Figure 3, illustrating that some clades, notably the orders Euphausiacea in crustaceans and Orthoptera in insects, have exceptionally large genomes that clearly stand out from the range of variation within other crustacean and insect groups. Comparing the genome size of the crustaceans and the insect reveal a larger variation in the former and smaller and more constant in the latter (Figure 3).

For the statistical associations between genome size and other variables, Blomberg's K only showed significant phylogenetic dependence (*K* > 1) for HAB for the crustaceans, while Pagel's λ indicated a correlation in the variation of C‐values, MDE and HAB and the phylogeny (λ ≈ 1, Table 1). For insects, in addition to C‐values, only DEV showed significant phylogenetic dependence (*K* > 1), with a variation corresponding to the dendrogram (λ ≈ 1, Table 1).

Regression models were evaluated for the crustaceans, with C‐values as the independent variable and maximum organism size (MOS), maximum absolute (most northern or southern) latitude (MAL), maximum depth (MDE), and habitat (HAB) as dependent variables (Table 2). Unsurprisingly, given the modest number of data for these categories, as well as the obvious problems in obtaining exact or representative data for the MOS, MAL, and MDE, it proved hard to arrive at strong statistical predictions. Regular linear optimal least square models (OLS/lm) revealed relatively low explanatory powers both with single and multivariate analyzes (*r* ^2^ < 0.10, Table 2). However, the OLS/lm of MDE, HAB, and multivariate MAL + MDE, could account for some of the C‐value variation seen in the dataset (*r* ^2^ > 0.10, Table 2). The OLS/lm regression coefficient (*b*) of MDE indicates a gradual increase in C‐values with maximum observed depth (<0.01 per meter, Table 2), consistent with the observation that the largest genomes among the marine crustaceans were observation in the deep‐water species. The coefficients of the multivariate MAL + MDE indicate a higher increase in C‐values with increasing maximum observed latitude for the marine crustaceans (0.13 per latitudinal degree, Table 2).

**Table 2:** Crustacean regression models

Independent variable:	C‐values				
OLS/lm	*b*	*r* ^2^	PGLS (lambda = ML)	*b*	*r* ^2^
**MOS** (*n* = 110)	<0.01	−0.01	**MOS**	<−0.01	<−0.01
**MOS + MAL** (*n* = 105)		0.02	**MOS + MAL**		0.06
MOS	<0.01		MOS	<−0.01	
MAL	0.08		MAL	0.11**	
**MAL** (*n* = 171)	0.07*	0.02	**MAL**	0.09**	0.04
**MAL + MDE** (*n* = 22)		0.23	**MAL + MDE**		0.28
MAL	0.13*		MAL	0.11*	
MDE	<0.01***		MDE	<0.01***	
**MDE** (*n* = 22)	<0.01***	0.16	**MDE**	<0.01***	0.24
**facHAB** (*n* = 182)		0.08	**facHAB**		0.02
Intercept	2.08*		Intercept	1.28	
facMarine	4.90***		facMarine	3.99*	
facTerrestrial	0.73		facTerrestrial	0.08	

*p < 0.05, **p < 0.01, ***p < 0.001.

Blomberg's *K* revealed a phylogenetic dependence of HAB (*K *> 1, Table 1) for the crustaceans analyzed, with most freshwater species belonging to Ostracoda, Branchiopoda, and cyclopoid copepods (Figure 2). Regression models using HAB can be adjusted for the taxonomic relationship using phylogenetic least squares (PGLS). The PGLS models revealed low fitting scores similar to what observed with OLS/lm (*r* ^2^ < 0.10, Table 2). As seen with OLS/lm, MDE and MAL + MDE may account for some of the C‐value variations seen in the dataset even after adjusting for phylogenetic relationships (*r* ^2^ = 0.24 and 0.28, Table 2).

The PGLS coefficient (*b*) of MDE and MAL + MDE was found to be similar to those of OLS/lm, The suggested phylogenetic‐dependent variable, HAB, was not found to account for much of the C‐value variation (*r* ^2^ < 0.02, Table 2), and the regression coefficients (*b*) were lowered after correcting for the taxonomy‐based phylogeny. The regression coefficients of both OLS/lm and PGLS (*b*) for HAB indicate larger expected genome sizes in marine species compared to freshwater and terrestrial species. However, one need to take into consideration that all cladocerans (with very small genomes), and most cyclopoids (also with rather small genomes) were freshwater species.

Regression models were evaluated for insects, with C‐values as the independent variable, and maximum absolute (most northern or southern latitude) (MAL), maximum elevation (MEL), and developmental mode (DEV) as dependent variables (Table 3). The OLS/lm models of the dependent variables revealed relatively low fitting scores both with single and multivariate analyzes (*r* ^2^ > 0.01, Table 3). The independent variable, C‐value, was found to be phylogenetic dependent (*K *> 1, Table 1), and all regression models were adjusted for the taxonomic relationship using PGLS. The PGLS models of insect C‐values revealed a similar pattern of low fitting scores as seen with the OLS/lm (*r* ^2^ < 0.10, Table 3). The phylogenetic‐dependent variable, DEV, was not found to account for much of the C‐value variation after correcting for the taxonomy‐based phylogeny (*r* ^2^ < 0.01, Table 3). The regression coefficients of PGLS (*b*) for DEV indicate larger expected genome sizes in insects with hemimetabolous development compared to those with ametabolous or holometabolous development.

**Table 3:** Insect regression models

Independent variable:	C‐values				
OLS/lm	*b*	*r* ^2^	PGLS (lambda = ML)	*b*	*r* ^2^
**MAL** (*n* = 432)	<0.01	<−0.01	**MAL**	<0.01	<−0.01
**MAL + MEL** (*n* = 323)		<−0.01	**MAL + MEL**		<−0.01
MAL	<0.01		MAL	<0.01	
MEL	<0.01		MEL	<−0.01	
**MEL** (*n *= 323)	<0.01	<−0.01	**MEL**	<−0.01	<−0.01
**facDEV** (*n* = 586)		0.17***	**facDEV**		<0.01
Intercept	1.66		Intercept	1.86	
facHemi.	0.78		facHemi.	2.22	
facHolo.	−1.12		facHolo.	1.69	

*p < 0.05, **p < 0.01, ***p < 0.001.

## 4 DISCUSSION

By contrasting these two major arthropod groups with respect to genome size, some striking differences in phylogenetic patterns become apparent, likely involving both proximate and ultimate drivers of genome size variation. The overall variability in genome size is less in insects than in crustaceans. As shown in previous studies (Gregory, 2002), most of this variation is found within the hemimetabolous insects. By comparison, the holometabolous insects have small genomes. However, as the latter is a monophyletic clade, it is difficult to disentangle phylogeny from developmental strategy as a driver of genome size in this context. For crustaceans, the picture is much more complex. Even though we found an effect of habitat, this may be confounded with phylogeny as most freshwater species of this database belong to the Cladocerans and cyclopoid copepods which has very small to small genomes. Moreover, there are striking differences in genome size between as well as within all marine groups, which suggest multiple causes for genome size variability. For some marine groups, the deep or cold‐water species also possessed the largest genomes in line with previous observations (Hessen et al., 2013; Rees et al., 2008; Timofeev, 2001), but not even for the crustaceans was any clear cline effect (latitude) or temperature effect observed. This does not mean that temperature (or high oxygen content correlated with low temperature) promotes larger genomes, but for obvious reasons it is impossible to arrive at precise data for geographical distribution or ambient temperature for the different species.

There are at least three explanations that all may provide different patterns of genome size. First, proximate mechanisms involve both whole‐genome duplication events and transposons proliferation or other structural traits of the genome itself. Second, large‐scale phylogenetic patterns may reflect maintenance of ancient traits due to low selective pressure for change, as well as common selective drivers in taxa with similar mode of life or habitat characteristics. Third, ambient drivers such as interactions between life history‐related parameters and the environment would tend to modify and obscure phylogenetic patterns.

Most evidence suggests that transposons proliferation is an important driver for genome size variation in arthropods. In insects, the species sequenced so far generally confirm a larger fraction of transposable and repetitive elements in large genomes (Maumus, Fiston‐Lavier, & Quesneville, 2015). Accordingly the smallest insect genome sequenced, in the Antarctic dipteran *Belgica antarctica*, has <1% transposons in its 0.1 pg genome (Kelley et al., 2014). By contrast, the 6.5 pg genome of the migratory locust *Locusta migratoria* contains >60% repeated elements (Wang et al., 2014), and likely is the major cause of the large genomes in the Orthoptera. However, this still cannot explain the entire difference in genome size of the two species, as even if excluding the repeated elements, the rest of the genome is 30 times larger in *L. migratoria*. In addition, related clades may also show striking gradients in fractions of transposons related to both body size and ambient conditions. This is clearly shown in the Drosophilidae, which range from 2.7% to 25% in the amount of transposable elements that correspond with genome size (Clark et al., 2007). However, within some insect clades, such as the beetle family Chrysomelinae, there are indications for chromosome duplication, with some species having 40−50 chromosomes and larger haploid genome size, while most others having about 20 chromosomes (Petitpierre, Segarra, & Juan, 1993). Relatively large genome size variation may also be observed on a smaller scale, even between small genomes. Thus, in ants, a relatively large genome size variation has been observed that is likely caused by gradual transposon accumulation as well as whole‐genome duplications (Tsutsui, Suarez, Spagna, & Johnston, 2008). Similarly in crustaceans, most evidence points toward transposons accumulation as the main source of bulky genomes, but the knowledge is limited owing to the scarcity of karyotypic information.

The proximate effect on genome size by transposons and genome duplications is likely affected by ultimate drivers such as phylogeny and the environment. Low temperature and slow developmental rates could, at least for the crustaceans, mean low selective pressure against transposons, effective population size may add to this (Lynch, 2010; Lynch & Conery, 2003; Lynch & Walsh, 2007). The population size argument is, however, most relevant for explaining the streamlined genomes of prokaryotes, and is less attributable to arthropods (i.e., locusts are among the insects that may attain largest populations, but still possess large genomes).

Suggestive correlations were found between genome size and proxies of environmental temperature (MAL, MAL+MDE, & MDE) for the crustaceans in this study (Table 2) both with (PGLS) and without phylogenetic contrast (OLS/lm). Contrary to the findings in amphipods species from Lake Baikal in Jeffery et al. (2016b), a phylogenetic structuring was observed for genome size variation in this study (λ ≈ 1), likely due to the use of a generalized phylogeny based on taxonomy (equal branch lengths) rather than transcriptome data (unequal branch lengths).

Both the insects and the crustaceans show potential for evolving large differences in genome size within closely related taxa as well as maintaining more clade‐specific genome size at different taxonomic levels (see Figure 3). The two arthropod groups display some striking differences in the structuring of the genome size variation, suggesting fundamental differences in selective drivers affecting the genome size. Such selective driver could be linked to habitat, that is a primarily terrestrial vs. aquatic mode of life. Accordingly, temperature often affects life history traits differently in the two environments, with strong diurnal and seasonal temperature fluctuation in terrestrial systems compared to the much more dampened variations in aquatic systems. These differences may be exemplified by patterns of adaptation to cold environments: Crustaceans, especially marine species, will experience relatively long‐growth season (several months), but with constantly low temperatures (Fox & Czesak, 2000; Huntley & Lopez, 1992). The fact that they frequently possess Bergmann clines with large body size and also large genomes is consistent with arguments for general cold adaptation (Hessen et al., 2013; Horne, Hirst, & Atkinson, 2017; Leinaas et al., 2016). By contrast, a main challenge for insects in cold environments is to cope with time limitation due to shorter growth seasons and a more stochastic climate. This may lead to a favoring of increased temperature‐specific growth rate, as well as reduced body size (Roff, 2002); that is converse Bergmann clines (Mousseau, 1997) like the Antarctic dipteran *Belgica antarctica*, with its dwarfed genome of 0.1 pg despite the cold habitat. Both of these adaptations will counteract increased genome size in cold environments. Thus, temperature may have less impact on the genome size in insects, which could possibly contribute to the general lower degree of variability in genome size than in the crustaceans. Metabolic rate and cell growth have been proposed to act as an ultimate driver of genome size evolution (Petrov, 2001). The observed effect of such fitness‐related traits has been suggested to break down when comparing groups above family level (Calatayud et al., 2016), these traits are probably of lesser importance explaining the genome size variation at higher taxonomic levels.

Developmental complexity has been suggested to be a main determinant of the differentiation of genome size between hemi‐ and holometabolous insects. Gregory (2002) suggested a threshold of approximately 2 pg haploid DNA per cell above which holometabolic metamorphosis becomes constrained by larger genomes. No mechanistic explanation is given, and the argument is challenged by some clear departures from this rule, notably within the Coleoptera (cf. Figure 2 and Hanrahan and Johnston (2011)). No support for this idea is seen in the crustaceans, where in fact the by far smallest genomes are found among the cladocerans with their simple direct development, while copepods with a complex development generally have much large genomes. It is possible that the strong structuring of the genome size by insect developmental mode is confounding the detection of other drivers (i.e., latitude or altitude, see Table 3), especially on such a large dataset.

In conclusion, some of the difference between insects and crustaceans likely reflect different life cycles in terrestrial versus aquatic habitats, but several ultimate drivers may operate depending on taxonomic resolution. Thus, a general expectation of increasing genome size along latitudinal gradients is not confirmed, and this is not only due to the aforementioned problems with obtained accurate information on range of distribution or temperature, but simply that genome size especially insects will be more sensitive to life cycle than temperature per se, or oxygen. For crustaceans, responses to latitude, depth, or temperature may be revealed by zooming in at finer taxonomic levels as demonstrated especially for amphipods and calanoid copepods (Leinaas et al., 2016; Rees et al., 2008).

The overall complexity in genome size and drivers thereof reflect the multiple proximate as well as ultimate drivers behind genome size. In addition, phylogenetic patterns in genome size may vary, depending on the taxonomic levels. While the major proximate cause of large genome size is transposon proliferation and/or whole‐genome duplication events, the relative role and relationship between these drivers are poorly explored in the arthropods. Also to what extent life history characteristics such as fast growth, complex developmental patterns, and parasitism may promote streamlined genomes, and mechanistically counteract intron proliferation is poorly understood. Given the major intrinsic role of genome size for fitness‐related phenotypic traits like cell size, body size, morphology, growth rate, behavior, life cycle, and potentially also speciation calls for a closer attention toward genome size as a phenotypic determinant.

## CONFLICT OF INTEREST

None declared.
